# *Leclercia adecarboxylata* Musculoskeletal Infection in an Immune Competent Pediatric Patient: An Emerging Pathogen?

**DOI:** 10.1155/2015/160473

**Published:** 2015-10-01

**Authors:** W. Jeffrey Grantham, Shawn S. Funk, Jonathan G. Schoenecker

**Affiliations:** ^1^Vanderbilt University Medical Center, MCE South Tower, Suite 4200, 1215 21st Avenue South, Nashville, TN 37232-8774, USA; ^2^Vanderbilt University Medical Center, 2200 Children's Way No. 11130, Nashville, TN 37232, USA

## Abstract

*Case*. An immune competent pediatric patient presented with a persistent lower extremity infection with *Leclercia adecarboxylata* after a penetrating injury. This case report details the presentation, clinical course, and treatment. *Conclusion*. *Leclercia adecarboxylata* has increasing reports in immunosuppressed and adult patients with musculoskeletal infection. This case now indicates that *Leclercia adecarboxylata* is a potential pathogen in immune competent children in musculoskeletal tissue.

## 1. Introduction

As a member of the Enterobacteriaceae family,* Leclercia adecarboxylata* is a motile Gram negative rod. First described in 1962, its description in the literature is isolated to case reports [1, 2].* Leclercia adecarboxylata* is regarded as normal gut flora for animals and has been isolated in human stool. The bacterium is primarily reported in immunosuppressed patients or polymicrobial infections [3]. There have been no case reports of* Leclercia adecarboxylata* in an immunocompetent musculoskeletal infection in a child. We present a case of foot infection associated with a foreign body by* Leclercia adecarboxylata* in an immunocompetent pediatric patient.

## 2. Case Report

A 9-year-old girl stepped on debris in a corn field while playing near a lake. She noted a penetrating injury to the left foot and presented to outside hospital where a foreign body was removed and was started on antibiotics. Foot pain persisted without systemic symptoms. Four days after the injury, pain persisted and she was taken to the operating suite for formal irrigation and debridement by an orthopaedic surgeon. The procedure removed a more substantial foreign body and a wick was placed in the wound. She completed three separate courses of antibiotics including cefalexin, sulfamethoxazole/trimethoprim, and cefepime for persistent drainage.

**Figure 1:**
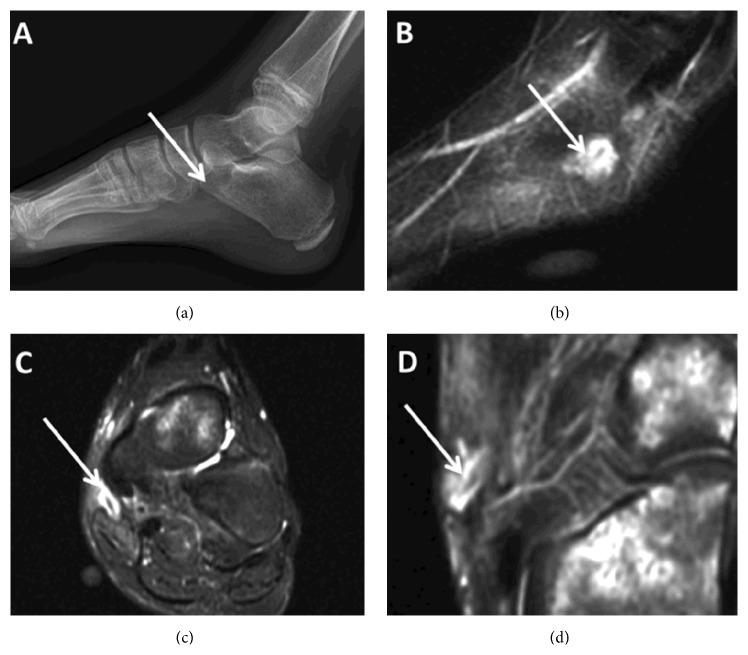
(a) X-ray and ((b)–(d)) sagittal, axial, and coronal MRI, respectively, indicating abscess with potential foreign body.

The patient presented to our institution two and one-half months after initial injury with continued pain and a small abscess at the medial midfoot. She was able to walk without a significant limp. Radiographs were negative for evidence of traumatic injury or foreign body (Figure 1(a)); however, MRI of the left foot had revealed a fluid collection surrounding a foreign body (Figures 1(b)–1(d)). Her laboratory markers were C-reactive protein 0.4 mg/L (normal 0.1–1.0 mg/L), erythrocytesedimentation rate 6 mm/hr (normal 0–20 mm/hr), and white blood cell count 6,800 cells/mcL (normal 4,000–13,200 cells/mcL).

**Figure 2:**
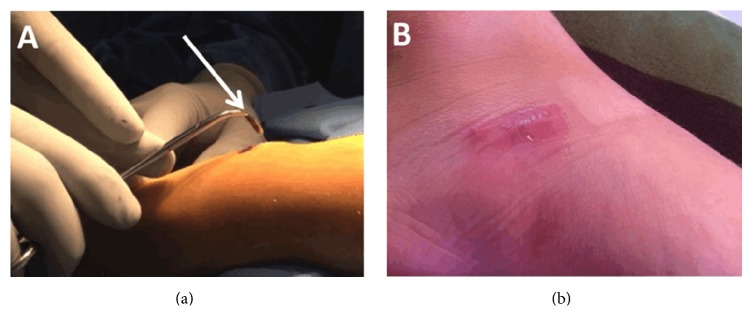
(a) Foreign body removal and (b) two-month follow-up.

She was taken to the operating room for irrigation and debridement. Aspiration was attempted with no significant purulence noted. The wound was explored and a 15 × 3 mm foreign body was removed (Figure 2(a)). The wound was irrigated, debrided, and closed primarily. Wound cultures were sent intraoperatively. The only organism isolated on blood and MacConkey agars was* Leclercia adecarboxylata* with susceptibility to multiple antibiotics and resistance to ampicillin. After consultation with pediatric infectious disease the patient was treated with a 14-day course of levofloxacin. Two months postoperatively there were no signs of continued infection or wound complications (Figure 2(b)). One year following her injury, she is without complaints.

## 3. Discussion

This case presents the first time* Leclercia adecarboxylata* has been isolated in an immunocompetent pediatric patient. Prior reports document this bacterium in immunocompromised patients [3–8]; however, recently there have been limited reports in immunocompetent patients [9, 10] in addition to the pediatric population [5, 8, 11]. Most cases report the bacteria with excellent susceptibility to antibiotics, as was the case for this isolate; however, resistant strains have been found [12]. Even catheter-associated infection with* Leclercia adecarboxylata* has been treated successfully with antibiotics alone without catheter removal [13]. In our case, the retained foreign body provided a medium for the persistent indolent infection despite courses of three different antibiotics. With removal of the foreign body the persistent infection resolved with routine oral antibiotic course. This bacterium, which previously had minimal recognition, now has increasing reports and is associated with pediatric immunocompetent patients with musculoskeletal infection. This report indicates that* Leclercia adecarboxylata* is a potential pathogen in immunocompetent children in musculoskeletal tissue.
